# Crystal structure of a hydrogen-bonded 2:1 co-crystal of 4-nitro­phenol and 4,4′-bi­pyridine

**DOI:** 10.1107/S205698902400971X

**Published:** 2024-10-08

**Authors:** Angela Gotingco, Merary Villanueva Contreras, Jeanette A. Wolfarth, S. Chantal E. Stieber, Zoe Y. Marr

**Affiliations:** a Department of Chemistry & Biochemistry California State Polytechnic University, Pomona 3801 W Temple Ave Pomona CA 91768 USA; Texas A & M University, USA

**Keywords:** crystal structure, co-crystal, hydrogen bonding

## Abstract

In the title compound, C_10_H_8_N_2_·2C_6_H_5_NO_3_, 4-nitro­phenol and 4,4′-bi­pyridine crystallized together in a 2:1 ratio in the space group *P*2_1_/*n*. There is a hydrogen-bonding inter­action between the nitro­gen atoms on the 4,4′-bi­pyridine mol­ecule and the hydrogen atom on the hydroxyl group on the 4-nitro­phenol, resulting in trimolecular units. This structure is a polymorph of a previously reported structure [Nayak & Pedireddi (2016 ▸). *Cryst. Growth Des.***16**, 5966–5975], which differs mainly due to a twist in the 4,4′-bi­pyridine mol­ecule.

## Abstract 2

4-Nitro­phenol and 4,4′-bi­pyridine crystallized together in a 2:1 ratio and in the space group *P*2_1_/*n*. There is a hydrogen-bonding inter­action between the nitro­gen atoms on the 4,4′-bi­pyridine mol­ecule and the hydrogen atom on the hydroxyl group on the 4-nitro­phenol resulting in trimolecular units.

## 1. Chemical context

Co-crystals are a growing field of science as crystalline solids can be engineered to have improved physical-chemical properties such as better solubility, stability, and bioavailability (Karimi-Jafari *et al.*, 2018 ▸). Co-crystals are defined as crystalline solids composed of two or more different mol­ecular and/or ionic compounds in a specific stoichiometric ratio and are neither solvates nor simple salts (Aitipamula *et al.*, 2012 ▸). They are held together by inter­molecular inter­actions such as hydrogen bonding, halogen bonding, and π–π stacking (Wang *et al.*, 2022 ▸). Generally, co-crystals are high yielding, making them an appealing candidate for crystal engineering in the realm of pharmaceutical purposes (Chettri *et al.*, 2024 ▸).
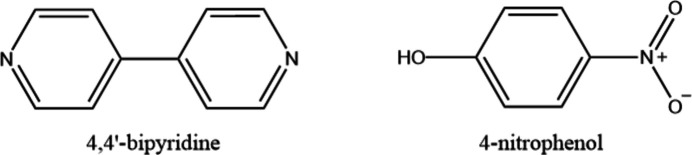


The chemicals used in this co-crystal consist of 4,4′-bi­pyridine and 4-nitro­phenol. 4,4′-Bi­pyridine can be found in a multitude of crystal structures due the pyridyl groups being suitable for both coordination polymers and co-crystals (Richard *et al.*, 2021 ▸). 4-Nitro­phenol is commonly found as a drug manufacturing and synthesis inter­mediate in the pharmaceutical industry. Specifically, this compound has been used in the production of compounds such as acetamino­phen, a drug used for pain relief, where it is nitrated and converted to 4-amino­phenol, an inter­mediate for acetamino­phenol (Abdollahi *et al.*, 2014 ▸). This is a highly appealing synthesis process for its greener approach to science in comparison to other inter­mediates used. Additionally, since 4-nitro­phenol takes on the color of a yellow crystalline solid, it is also a suitable candidate that is used to produce pigments/dyes such as leather darkener (National Center for Biotechnology Information, 2024 ▸).

## 2. Structural commentary

**Figure 1:**
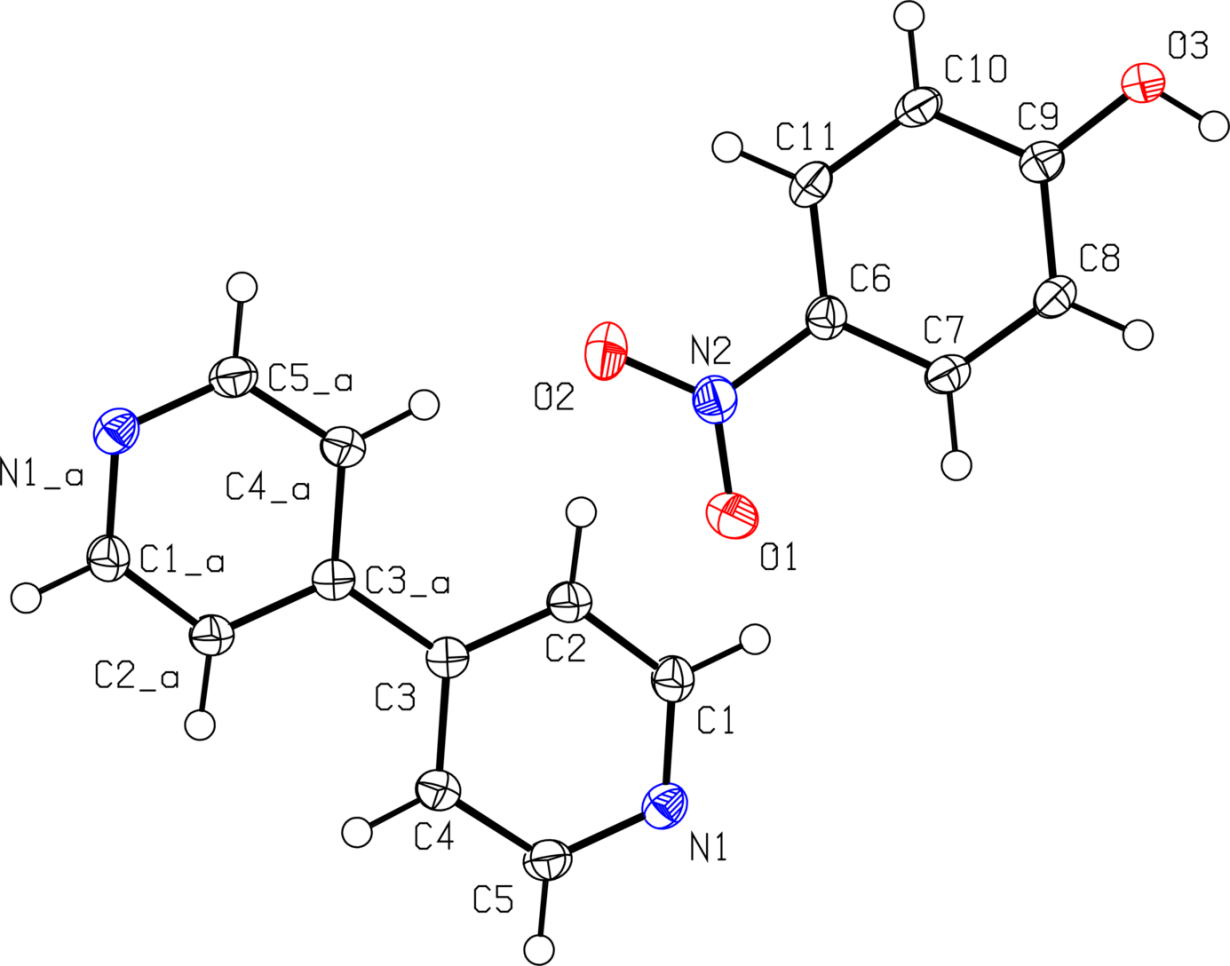
A view of the structure containing 4,4′-bi­pyridine and 4-nitro­phenol showing the atom-labeling scheme. Atoms that contain _a in the label are symmetry generated (−*x* + 2, − *y* + 1, −*z*). The displacement ellipsoids are drawn at 50% probability.

4,4′-Bi­pyridine and 4-nitro­phenol co-crystallized in a 1:2 ratio with the asymmetric unit containing one mol­ecule of 4-nitro­phenol and half of a 4,4′-bi­pyridine mol­ecule in the *P*2_1_/*n* space group. Half of the atoms from the 4,4′-bi­pyridine mol­ecule sit on Wyckoff position 4*e*, and the other half is generated by the center of inversion (0, 1/2, 0) (Fig. 1 ▸). The nitro groups exhibit a trigonal–planar geometry with bond angles of 122.44 (16)° for O1—N2—O2, 118.51 (14)° for O1—N2—C6, and 119.06 (15) for O2—N2—C6. The N—O/N=O bonds have lengths of 1.232 (2) and 1.2346 (19) Å, which are in between the average values for N—O and N=O bonds, as expected due to resonance. The mol­ecular geometry of the hydroxyl group is bent with with an angle of 110.4 (19)° for H3—O3—C9. The aromatic benzene ring has bond angles ranging between 119.32 (15) and 121.33 (15)°, as expected for *sp*^2^-hybridized carbons.

## 3. Supra­molecular features

**Figure 2:**
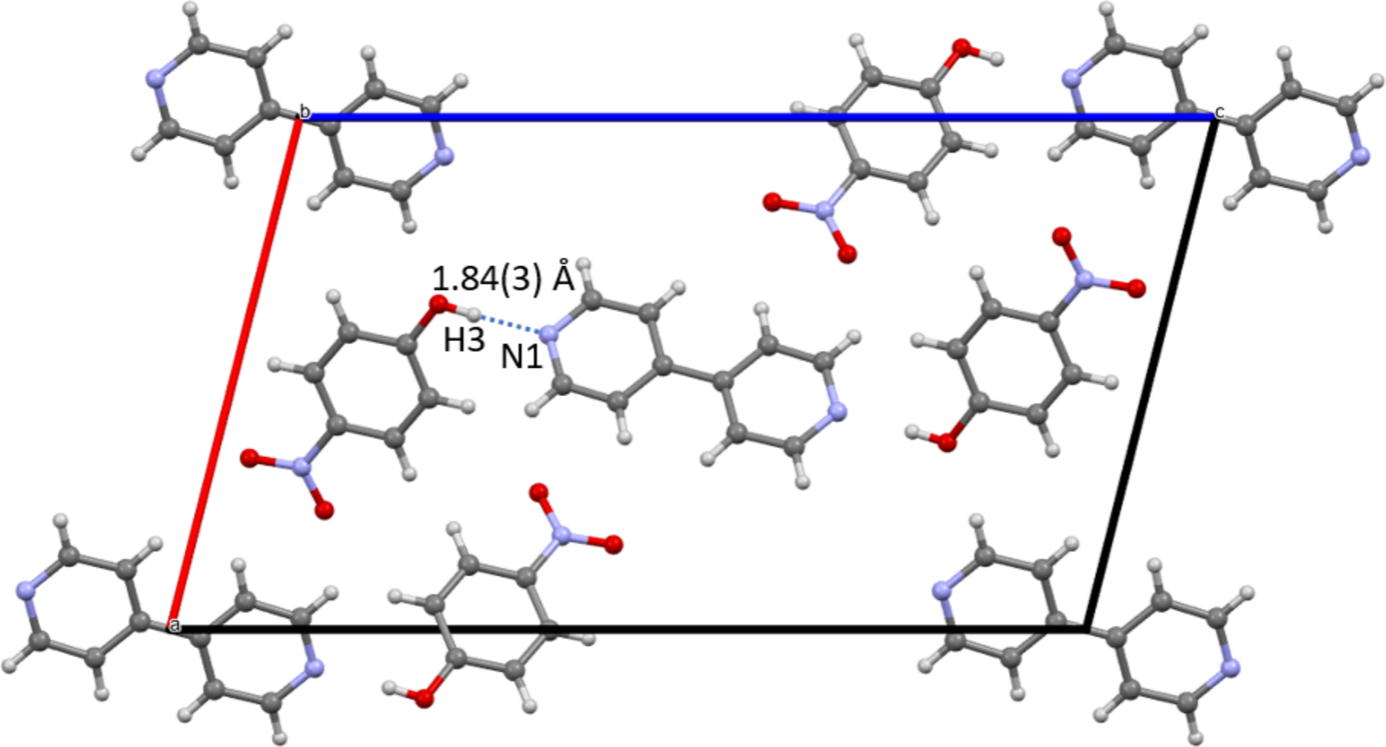
Diagram of the packed unit cell highlighting the hydrogen-bonding inter­action between the hydrogen on the 4-nitro­phenol and the nitro­gen atom on the 4,4′-bi­pyridine. View down [010].

In the structure, each nitro­gen on the 4,4′-bi­pyridine is hydrogen bonded to the hydroxyl group from the 4-nitro­phenol, which results in the formation of trimolecular units that propagate along [001] (Fig. 2 ▸). There is a hydrogen-bonding inter­action (Table 1 ▸) between the two mol­ecules with a *D*⋯*A* distance between H3 and N1 of 1.84 (3) Å. In addition to hydrogen bonding, there are also π–π inter­actions between the ring systems of the adjacent 4,4′-bi­pyridine mol­ecules having a plane centroid to plane centroid distance of 3.8255 (11) Å.

## 4. Database survey

The crystal structure that is reported herein is a polymorph of a co-crystal (refcode AWEVUV) published by Nayak & Pedireddi (2016 ▸). Similar synthesis and crystallization conditions were used but there are slight differences in the structure. The asymmetric unit of the previous structure contains three mol­ecules, two 4-nitro­phenol and one 4,4′-bi­pyridine, and the 4,4′-bi­pyridine does not lie on a symmetry operation. In the previously published structure, the pyridyl groups are rotated about the C13—C22 bond with a plane twist angle of 24.60 (8)° whereas the pyridyl rings sit in plane with one another in the structure reported above. The unit-cell parameters and space group for the two structures also differ (Table 2 ▸).

## 5. Synthesis and crystallization

The synthesis for the newly reported co-crystal is modified from the procedure published by Nayak & Pedireddi (2016 ▸) with a 2:1 rather than a 1:1 ratio of 4,4′-bi­pyridine and 4-nitro­phenol used. In addition, the method of heating was changed from a warm water bath to gentle heating directly on a hot plate. These differences in the synthesis could contribute to the deviation of the packing of the mol­ecules from the original structure.

A 2:1 molar ratio of 4,4′-bi­pyridine and 4-nitro­phenol was used to synthesize the new co-crystal. 20.0 mg (0.128 mmol, 2 eq) of 4,4′-bi­pyridine and 8.9 mg (0.0640 mmol, 1 eq) of 4-nitro­phenol were added to a 20 mL scintillation vial. 4.4 mL of methanol was added to the vial and then the solution was warmed up on a hot plate to dissolve the solids. Once fully dissolved, the solution was cooled to room temperature. The sample underwent slow evaporation and to control evaporation rate, small holes were punctured on the parafilm covering as the solution was left to evaporate for 2 weeks. The resulting crystals were clear, colorless prisms. These crystals were grown as part of class CHM 5720 ‘Current advances in Inorganic Chemistry – Introduction to Crystallography’ at Cal Poly Pomona.

## 6. Refinement

Crystal data, data collection and structure refinement details are summarized in Table 3 ▸. All hydrogens except H3 were placed at calculated positions using AFIX commands and refined using a riding model. The position of H3 was determined using the Fourier difference map and refined freely.

## Supplementary Material

Crystal structure: contains datablock(s) I. DOI: 10.1107/S205698902400971X/jy2052sup1.cif

Structure factors: contains datablock(s) I. DOI: 10.1107/S205698902400971X/jy2052Isup3.hkl

Supporting information file. DOI: 10.1107/S205698902400971X/jy2052Isup3.cml

CCDC reference: 2388458

Additional supporting information:  crystallographic information; 3D view; checkCIF report

## Figures and Tables

**Table 1:** Hydrogen-bond geometry (Å, °)

*D*—H⋯*A*	*D*—H	H⋯*A*	*D*⋯*A*	*D*—H⋯*A*
O3—H3⋯N1^i^	0.86 (3)	1.84 (3)	2.6921 (19)	174 (3)

Symmetry code: (i) 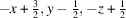.

**Table 2:** Comparison of unit-cell parameters (Å, °)

Parameter	Nayak & Pedireddi (2016 ▸)	Title compound
Crystal system	Monoclinic	Monoclinic
Space group	*P*2_1_/*c*	*P*2_1_/*n*
*a*	19.090 (4)	12.3711 (7)
*b*	3.8080 (10)	3.8255 (2)
*c*	27.3470 (10)	21.4175 (12)
*β*	98.38 (3)	104.195 (2)

**Table 3:** Experimental details

Crystal data	
Chemical formula	C_10_H_8_N_2_·2C_6_H_5_NO_3_
*M*_r_	434.40
Crystal system, space group	Monoclinic, *P*2_1_/*n*
Temperature (K)	123
*a*, *b*, *c* (Å)	12.3711 (7), 3.8255 (2), 21.4175 (12)
β (°)	104.195 (2)
*V* (Å^3^)	982.65 (9)
*Z*	2
Radiation type	Mo *K*α
μ (mm^−1^)	0.11
Crystal size (mm)	0.47 × 0.07 × 0.03

Data collection	
Diffractometer	Bruker D8 Venture
Absorption correction	Multi-scan (*SADABS*; Krause *et al.*, 2015 ▸)
*T*_min_, *T*_max_	0.501, 0.746
No. of measured, independent and observed [*I* > 2σ(*I*)] reflections	24119, 2957, 2302
*R*_int_	0.076
(sin θ/λ)_max_ (Å^−1^)	0.711

Refinement	
*R*[*F*^2^ > 2σ(*F*^2^)], *wR*(*F*^2^), *S*	0.057, 0.161, 1.10
No. of reflections	2957
No. of parameters	149
H-atom treatment	H atoms treated by a mixture of independent and constrained refinement
Δρ_max_, Δρ_min_ (e Å^−3^)	0.47, −0.37

Computer programs: *APEX4* and *SAINT* V8.40B (Bruker, 2018 ▸), *SHELXT2018/2* (Sheldrick, 2015*a* ▸), *SHELXL* (Sheldrick, 2015*b* ▸) and *OLEX2* (Dolomanov *et al.*, 2009 ▸).

## supplementary crystallographic information

### 4,4'-Bipyridine–4-nitrophenol (1/2) Crystal data

**Table:**

C_10_H_8_N_2_·2C_6_H_5_NO_3_	*F*(000) = 452
*M**_r_* = 434.40	*D*_x_ = 1.468 Mg m^−^^3^
Monoclinic, *P*2_1_/*n*	Mo *K*α radiation, λ = 0.71073 Å
*a* = 12.3711 (7) Å	Cell parameters from 5585 reflections
*b* = 3.8255 (2) Å	θ = 2.2–30.3°
*c* = 21.4175 (12) Å	µ = 0.11 mm^−^^1^
β = 104.195 (2)°	*T* = 123 K
*V* = 982.65 (9) Å^3^	Prism, clear colourless
*Z* = 2	0.47 × 0.07 × 0.03 mm

### 4,4'-Bipyridine–4-nitrophenol (1/2) Data collection

**Table:**

Bruker D8 Venture diffractometer	2302 reflections with *I* > 2σ(*I*)
φ and ω scans	*R*_int_ = 0.076
Absorption correction: multi-scan (SADABS; Krause *et al.*, 2015)	θ_max_ = 30.4°, θ_min_ = 2.0°
*T*_min_ = 0.501, *T*_max_ = 0.746	*h* = −17→17
24119 measured reflections	*k* = −5→5
2957 independent reflections	*l* = −30→30

### 4,4'-Bipyridine–4-nitrophenol (1/2) Refinement

**Table:**

Refinement on *F*^2^	Primary atom site location: dual
Least-squares matrix: full	Hydrogen site location: mixed
*R*[*F*^2^ > 2σ(*F*^2^)] = 0.057	H atoms treated by a mixture of independent and constrained refinement
*wR*(*F*^2^) = 0.161	*w* = 1/[σ^2^(*F*_o_^2^) + (0.0648*P*)^2^ + 0.7817*P*] where *P* = (*F*_o_^2^ + 2*F*_c_^2^)/3
*S* = 1.10	(Δ/σ)_max_ < 0.001
2957 reflections	Δρ_max_ = 0.47 e Å^−^^3^
149 parameters	Δρ_min_ = −0.37 e Å^−^^3^
0 restraints	

### 4,4'-Bipyridine–4-nitrophenol (1/2) Special details

**Table:**

Geometry. All esds (except the esd in the dihedral angle between two l.s. planes) are estimated using the full covariance matrix. The cell esds are taken into account individually in the estimation of esds in distances, angles and torsion angles; correlations between esds in cell parameters are only used when they are defined by crystal symmetry. An approximate (isotropic) treatment of cell esds is used for estimating esds involving l.s. planes.

### 4,4'-Bipyridine–4-nitrophenol (1/2) Fractional atomic coordinates and isotropic or equivalent isotropic displacement parameters (Å^2^)

**Table:**

	*x*	*y*	*z*	*U*_iso_*/*U*_eq_	
O3	0.36469 (10)	0.2681 (4)	0.20323 (6)	0.0241 (3)	
O1	0.76718 (11)	1.0265 (4)	0.13543 (7)	0.0315 (3)	
O2	0.66554 (13)	0.9310 (5)	0.03941 (6)	0.0372 (4)	
N1	1.07530 (12)	0.6778 (4)	0.16823 (7)	0.0224 (3)	
N2	0.68271 (12)	0.9075 (4)	0.09853 (7)	0.0243 (3)	
C3	1.01552 (13)	0.5393 (4)	0.03500 (7)	0.0184 (3)	
C6	0.60089 (14)	0.7357 (4)	0.12575 (8)	0.0196 (3)	
C11	0.49848 (14)	0.6398 (5)	0.08606 (8)	0.0220 (3)	
H11	0.482527	0.682639	0.041035	0.026*	
C7	0.62601 (14)	0.6748 (4)	0.19178 (8)	0.0209 (3)	
H7	0.696365	0.741876	0.218231	0.025*	
C1	0.97805 (15)	0.5281 (5)	0.13953 (8)	0.0227 (4)	
H1	0.928687	0.468694	0.165566	0.027*	
C9	0.44362 (14)	0.4190 (4)	0.17947 (8)	0.0202 (3)	
C8	0.54795 (14)	0.5162 (4)	0.21849 (8)	0.0211 (3)	
H8	0.564758	0.472398	0.263503	0.025*	
C2	0.94480 (14)	0.4545 (5)	0.07449 (8)	0.0223 (3)	
H2	0.874671	0.347421	0.056842	0.027*	
C10	0.42023 (14)	0.4814 (5)	0.11295 (8)	0.0228 (4)	
H10	0.350120	0.414291	0.086214	0.027*	
C5	1.14324 (14)	0.7645 (5)	0.13058 (8)	0.0238 (4)	
H5	1.212254	0.874593	0.149718	0.029*	
C4	1.11678 (14)	0.6999 (5)	0.06463 (8)	0.0230 (4)	
H4	1.167421	0.764784	0.039720	0.028*	
H3	0.386 (2)	0.254 (8)	0.2445 (15)	0.053 (8)*	

### 4,4'-Bipyridine–4-nitrophenol (1/2) Atomic displacement parameters (Å^2^)

**Table:**

	*U*^11^	*U*^22^	*U*^33^	*U*^12^	*U*^13^	*U*^23^
O3	0.0195 (6)	0.0320 (7)	0.0194 (6)	−0.0049 (5)	0.0022 (5)	0.0017 (5)
O1	0.0236 (6)	0.0378 (8)	0.0325 (7)	−0.0049 (6)	0.0056 (5)	0.0044 (6)
O2	0.0364 (8)	0.0536 (10)	0.0226 (7)	−0.0013 (7)	0.0094 (6)	0.0087 (6)
N1	0.0234 (7)	0.0230 (7)	0.0203 (7)	0.0029 (6)	0.0044 (5)	−0.0015 (5)
N2	0.0222 (7)	0.0275 (8)	0.0238 (7)	0.0040 (6)	0.0066 (5)	0.0048 (6)
C3	0.0183 (7)	0.0169 (7)	0.0198 (7)	0.0014 (6)	0.0044 (6)	−0.0002 (6)
C6	0.0201 (7)	0.0202 (7)	0.0187 (7)	0.0029 (6)	0.0052 (6)	0.0017 (6)
C11	0.0240 (8)	0.0252 (8)	0.0153 (7)	0.0043 (7)	0.0018 (6)	0.0005 (6)
C7	0.0195 (7)	0.0220 (8)	0.0185 (7)	−0.0005 (6)	−0.0004 (6)	0.0004 (6)
C1	0.0234 (8)	0.0245 (8)	0.0213 (8)	0.0000 (6)	0.0077 (6)	−0.0009 (6)
C9	0.0196 (7)	0.0202 (8)	0.0196 (7)	0.0015 (6)	0.0028 (6)	−0.0009 (6)
C8	0.0232 (8)	0.0223 (8)	0.0159 (7)	−0.0005 (6)	0.0009 (6)	0.0003 (6)
C2	0.0200 (8)	0.0254 (8)	0.0212 (8)	−0.0030 (6)	0.0044 (6)	−0.0024 (6)
C10	0.0200 (8)	0.0279 (9)	0.0175 (7)	0.0010 (6)	−0.0010 (6)	−0.0015 (6)
C5	0.0190 (8)	0.0283 (9)	0.0227 (8)	0.0002 (6)	0.0023 (6)	−0.0025 (7)
C4	0.0182 (7)	0.0283 (9)	0.0224 (8)	−0.0015 (6)	0.0048 (6)	−0.0011 (7)

### 4,4'-Bipyridine–4-nitrophenol (1/2) Geometric parameters (Å, º)

**Table:**

O3—C9	1.338 (2)	C11—C10	1.382 (2)
O3—H3	0.86 (3)	C7—H7	0.9500
O1—N2	1.232 (2)	C7—C8	1.378 (2)
O2—N2	1.2346 (19)	C1—H1	0.9500
N1—C1	1.338 (2)	C1—C2	1.381 (2)
N1—C5	1.341 (2)	C9—C8	1.405 (2)
N2—C6	1.444 (2)	C9—C10	1.403 (2)
C3—C3^i^	1.484 (3)	C8—H8	0.9500
C3—C2	1.396 (2)	C2—H2	0.9500
C3—C4	1.400 (2)	C10—H10	0.9500
C6—C11	1.391 (2)	C5—H5	0.9500
C6—C7	1.391 (2)	C5—C4	1.392 (2)
C11—H11	0.9500	C4—H4	0.9500
			
C9—O3—H3	110.4 (19)	C2—C1—H1	117.9
C1—N1—C5	117.07 (15)	O3—C9—C8	122.52 (15)
O1—N2—O2	122.44 (16)	O3—C9—C10	118.15 (15)
O1—N2—C6	118.51 (14)	C10—C9—C8	119.32 (15)
O2—N2—C6	119.06 (15)	C7—C8—C9	120.32 (15)
C2—C3—C3^i^	121.30 (18)	C7—C8—H8	119.8
C2—C3—C4	116.84 (15)	C9—C8—H8	119.8
C4—C3—C3^i^	121.86 (18)	C3—C2—H2	120.4
C11—C6—N2	119.80 (15)	C1—C2—C3	119.19 (16)
C11—C6—C7	121.33 (15)	C1—C2—H2	120.4
C7—C6—N2	118.86 (15)	C11—C10—C9	120.44 (15)
C6—C11—H11	120.4	C11—C10—H10	119.8
C10—C11—C6	119.16 (15)	C9—C10—H10	119.8
C10—C11—H11	120.4	N1—C5—H5	118.7
C6—C7—H7	120.3	N1—C5—C4	122.70 (16)
C8—C7—C6	119.41 (15)	C4—C5—H5	118.7
C8—C7—H7	120.3	C3—C4—H4	120.0
N1—C1—H1	117.9	C5—C4—C3	119.98 (16)
N1—C1—C2	124.22 (16)	C5—C4—H4	120.0
			
O3—C9—C8—C7	−179.23 (16)	C3^i^—C3—C4—C5	−179.13 (19)
O3—C9—C10—C11	179.35 (16)	C6—C11—C10—C9	0.2 (3)
O1—N2—C6—C11	171.57 (16)	C6—C7—C8—C9	−0.4 (3)
O1—N2—C6—C7	−7.4 (2)	C11—C6—C7—C8	0.0 (3)
O2—N2—C6—C11	−8.2 (2)	C7—C6—C11—C10	0.1 (3)
O2—N2—C6—C7	172.86 (16)	C1—N1—C5—C4	−1.0 (3)
N1—C1—C2—C3	−0.1 (3)	C8—C9—C10—C11	−0.6 (3)
N1—C5—C4—C3	0.2 (3)	C2—C3—C4—C5	0.6 (3)
N2—C6—C11—C10	−178.82 (16)	C10—C9—C8—C7	0.7 (3)
N2—C6—C7—C8	178.95 (15)	C5—N1—C1—C2	0.9 (3)
C3^i^—C3—C2—C1	179.06 (19)	C4—C3—C2—C1	−0.7 (2)

Symmetry code: (i) −*x*+2, −*y*+1, −*z*.

### 4,4'-Bipyridine–4-nitrophenol (1/2) Hydrogen-bond geometry (Å, º)

**Table:**

*D*—H···*A*	*D*—H	H···*A*	*D*···*A*	*D*—H···*A*
O3—H3···N1^ii^	0.86 (3)	1.84 (3)	2.6921 (19)	174 (3)

Symmetry code: (ii) −*x*+3/2, *y*−1/2, −*z*+1/2.
